# The role of HDAC3 in inflammation: mechanisms and therapeutic implications

**DOI:** 10.3389/fimmu.2024.1419685

**Published:** 2024-07-10

**Authors:** Noah Watson, Sivaraman Kuppuswamy, William Luke Ledford, Sangeetha Sukumari-Ramesh

**Affiliations:** Department of Pharmacology and Toxicology, Medical College of Georgia, Augusta University, Augusta, GA, United States

**Keywords:** HDAC3, inflammation, histone deacetylases, epigenetic mechanisms, HDAC

## Abstract

Histone deacetylases (HDACs) are critical regulators of inflammatory gene expression, and the efficacy of pan-HDAC inhibitors has been implicated in various disease conditions. However, it remains largely unclear how HDACs precisely regulate inflammation. To this end, evaluating the isoform-specific function of HDACs is critical, and the isoform-specific targeting could also circumvent the off-target effects of pan-HDAC inhibitors. This review provides an overview of the roles of HDAC3, a class I HDAC isoform, in modulating inflammatory responses and discusses the molecular mechanisms by which HDAC3 regulates inflammation associated with brain pathology, arthritis, cardiovascular diseases, lung pathology, allergic conditions, and kidney disorders. The articles also identify knowledge gaps in the field for future studies. Despite some conflicting reports, the selective inhibition of HDAC3 has been demonstrated to play a beneficial role in various inflammatory pathologies. Exploring the potential of HDAC3 inhibition to improve disease prognosis is a promising avenue requiring further investigation.

## Introduction

Histone acetylation is a post-translational histone modification that regulates diverse functions, such as protein-protein interactions, DNA recognition, protein stability, and gene expression (1, 2). The interplay between histone acetyltransferases (HATs) and histone deacetylases (HDACs) dynamically modulates the acetylation status of histones, resulting in structural changes to chromatin and thus transcriptional regulation (1, 2). Enhanced HAT activity facilitates chromatin relaxation and gene transcription (3–5), whereas HDACs remove acetyl groups from histones, causing chromatin condensation and gene repression (6). In line with this, the inhibition of HDAC activity causes histone hyperacetylation and transcriptional activation of genes. DNA expression microarrays indicate that the effect of HDAC inhibitors [HDACi (s)] on gene expression is not global but rather limited to a subset of genes (1–7%) (7–11).

HDACs are a family of proteins, with 18 HDAC isoforms currently identified in humans (12). Based on the sequence homology, HDACs are classified into class I (HDAC1, 2, 3 and 8), class IIa (HDAC4, 5, 7 and 9), class IIb (HDAC6 and 10), class III (SIRT1–7) and class IV (HDAC11). The enzymatic activity of class I, II, and IV HDACs requires zinc metal, whereas sirtuins or class III require nicotine adenine dinucleotide as a co-factor (13). Class I HDACs are primarily nuclear and play an important role in cell survival, apoptosis, proliferation, and differentiation (14, 15). Class II HDACs are found in the nucleus and cytoplasm (16, 17). Besides deacetylating histones, HDACs can modulate the acetylation status of nuclear, cytosolic, and mitochondrial non-histone proteins (18), including transcription factors, affecting their structure, stability, interactions, function, and signaling. Hence, HDACs can regulate a wide range of cellular processes.

HDACs have earned much attention in the field of immunology, as they are implicated in various innate and adaptive immune responses, including the synthesis and release of cytokines (19, 20). To this end, the acetylation status of histones and non-histone proteins can affect the expression of inflammatory genes. Also, broad-spectrum HDAC inhibitors exert anti-inflammatory effects and reduce inflammation and disease severity in a wide range of conditions (21). Though HDACs are attractive targets due to the existing clinical applicability of HDAC inhibitors in various disorders (22–24), the use of pan-HDAC inhibitors in clinical trials is associated with several adverse effects, such as fatigue, nausea/vomiting, and diarrhea (25). To alleviate the unwanted side effects of pan-HDAC inhibition, emerging research focuses on targeting individual isoforms of HDACs. The selective targeting of isoforms will also help elucidate the precise mechanism by which HDACs regulate diverse disease processes, which remains largely unclear and controversial.

Histone deacetylase 3 (HDAC3), a class I HDAC isoform, is unique among class I HDACs as it carries nuclear export and localization signals and can shuttle between the nucleus and cytoplasm (26). Owing partly to its non-nuclear localization, HDAC3 acts beyond as a co-repressor (27). Also, HDAC3 exerts enzymatic and non-enzymatic functions. The enzymatic activity of HDAC3 is an important mechanism regulating gene transcription. HDAC3 has often been purified as part of a complex that contains a co-repressor, NCoR1 (nuclear receptor co-repressor), or its homolog NCoR2 (SMRT; silencing mediator of retinoic and thyroid receptors) (28–30). HDAC3 is the prominent HDAC associated with NCoR1 and SMRT (31, 32), which regulates transcriptional repression. Hence, HDAC3 could have distinct functions compared to other class I HDACs. HDAC3 requires interaction with the deacetylase activating domain (DAD) within SMRT or NCoR1 for its enzymatic activity (33). It has been documented that inositol tetraphosphate facilitates the interaction between HDAC3 and DAD (34). Binding to inositol tetraphosphate and DAD triggers a conformational change in HDAC3, allowing substrates to access the catalytic site (34, 35). Global deletion of HDAC3 is embryonically lethal, but mice with mutations in the DAD of both NCoR1 and SMRT live to adulthood despite undetectable deacetylase activity in the embryo (36, 37) suggesting that non-enzymatic activity of HDAC3 drives the growth or survival of embryos.

There is a strong body of research connecting HDAC3 to the inflammatory response. Therefore, evaluating the therapeutic efficacy of HDAC3 inhibition in various inflammatory disease contexts is an ongoing and emerging area of research interest. Functionally, HDAC3 is crucial for the induction of pro-inflammatory gene expression in macrophages in response to inflammatory stimulus, lipopolysaccharide (38). The anti-inflammatory cytokine-mediated stimulation of macrophages into alternate activation involves epigenetic mechanisms (39, 40), and macrophages lacking HDAC3 are phenotypically similar to IL-4-induced alternatively activated macrophages (39). Also, HDAC3 is demonstrated to mediate mitochondrial adaptations to drive IL-1β dependent inflammation in macrophages through non-histone deacetylation (41). Besides, HDAC3 expression was found to be upregulated during various inflammatory settings (38, 42, 43), and selective HDAC3 inhibition modulates inflammation in multiple pathologies. Herein, we provide an overview of the functional roles of HDAC3 regarding inflammation associated with various disease conditions.

## HDAC3 in neuroinflammation

Neuroinflammation, the inflammatory response in the CNS, is characterized by glial activation, and upregulation, and secretion of inflammatory mediators such as cytokines, chemokines, and reactive oxygen species. The degree of neuroinflammation depends on the type, duration, and severity of insult or injury. In the uninjured brain, microglia, the inflammatory cells of the CNS, actively survey the brain microenvironment for non-functional neurons and serve as the sentinels of infection. Upon a brain insult or injury, microglia undergo activation, resulting in transcriptional and phenotypical changes with the release of various cytokines, chemokines, and reactive oxygen species. Activated microglia/macrophages polarize to a pro-inflammatory M1 phenotype or an anti-inflammatory M2 phenotype (44) and exhibit migratory and phagocytic potential, contributing to disease progression or repair. Microglia respond to both systemic and brain pathologies. Neuroinflammation also often results in the recruitment of peripheral cells to the brain, further aggravating or alleviating the neuroinflammatory cascade depending on the stage or type of neuropathological condition. In general, the microglia-mediated immune response or the transient activation of microglia is regarded as an intrinsic mechanism to protect or repair the brain. However, neuropathological conditions often result in chronic activation of microglia, culminating in neuronal death, neurodegeneration, and neurological and cognitive decline.

Growing evidence suggests that HDAC3 could be a potential target to modulate neuroinflammation. For instance, the use of a broad-spectrum HDAC inhibitor, valproic acid, modulated microglial polarization towards M2 phenotype and improved outcomes post-traumatic spinal cord injury (45). Valproic acid-mediated neuroprotection was associated with the inhibition of HDAC3 expression and activity in the lesioned spinal cord as well as upregulation of STAT1 - NF-κB P65 interaction, thereby attenuating NF-κB P65 DNA binding (45). As per the study, HDAC3 could serve as a critical modulator of STAT1 - NF-κB P65 interaction and neuroinflammation by regulating the acetylation status of both STAT1 and NF-κB P65 (45). Also, in ischemia/reperfusion-induced brain injury, HDAC3-mediated regulation of NF-κB p65 acetylation in microglia has been demonstrated to play a role in neuroinflammation (46). Mechanistically, it was demonstrated that HDAC3-mediated cGAS transcription and neuroinflammation in ischemia/reperfusion-induced brain injury were associated with NF-κB p65 deacetylation (46). Furthermore, a pan-HDAC inhibitor, Belinostat, attenuated neuroinflammation in an experimental model of autoimmune encephalomyelitis by increasing the acetylation status of NF-κB P65 with a reduction in the expression of HDAC3 (47). In line with this observation, a selective inhibitor of HDAC3, RGFP966, reduced demyelination in a cuprizone-induced demyelination model and improved neurological behavior (48). The study also showed that RGFP966 significantly reduced M1-like microglia/macrophage activation and the levels of proinflammatory cytokines, such as TNF-α, IL-1β, and iNOS. The neuroprotection conferred by RFGP966 in the mouse model of cuprizone was attributed to modulating P2X7R/STAT3/NF-κB p65/NLRP3 signaling pathways (48). Another study documented an increased HDAC3 expression in microglia/macrophages in a mouse model of ischemic stroke and RGFP966-mediated reduction in brain damage by attenuating AIM2 expression, possibly via modulating STAT1 acetylation (49). Though RGFP966 is being widely used for selectively targeting HDAC3, a study used BRD3308 to selectively inhibit HDAC3 in a mouse model after intraventricular hemorrhage. BRD3308 reduced neuroinflammation and microglial pyroptosis, with the modulation of the PPARγ/NLRP3/GSDMD pathway after intraventricular hemorrhage (50).

Consistent with the role of HDAC3 in neuroinflammatory responses, HDAC3 inhibition attenuated the expression of proinflammatory cytokines in repeatedly LPS-challenged human monocytes and M1 macrophages (51). Also, HDAC3 inhibition using RGFP966 reduced LPS-induced primary microglial activation (52). In the presence of LPS, RGFP966 modulated the expression of proteins involved in the TLR pathway and the phosphorylation of STAT3 and STAT5 in primary microglia (52). Taken together, the data indicate a crucial role for HDAC3 in regulating the acetylation status of transcription factors such as NF-κB p65, STAT1, and STAT3 and, hence, neuroinflammation.

## HDAC3 in arthritic inflammation

Rheumatoid arthritis (RA) is an autoimmune disease characterized by a chronic state of unknown etiology and progressive damage to the cartilage that can lead to lifelong disability (53). Dysregulated immune function is a contributing factor in RA pathogenesis and disease progression. Both local and systemic immune abnormalities occur in association with RA (54). During the course of the disease, fibroblast-like synoviocytes (FLS) exhibit abnormal activation, which is associated with altered expression of major histocompatibility complex (MHC)-II, pro-inflammatory cytokines, adhesion molecules, proangiogenic factors, and matrix-degrading enzymes (55–57), contributing to synovial inflammation and joint damage (58). Furthermore, the pathogenesis of RA is closely related to the abnormal activation of FLS (59).

Histone deacetylases play roles in RA progression, and HDAC inhibitors exhibited therapeutic efficacy and anti-inflammatory effects in animal models of RA (60–65). A study showed that the pan-HDAC inhibitors, Trichostatin A and ITF2357, suppressed IL-6 production induced by IL-1β, TNF-α, and TLR ligands in RA-FLS (66). Moreover, a class I HDAC inhibitor, MS-275, and a pan HDAC inhibitor, Suberoylanilide Hydroxamic Acid, attenuated the inflammatory response in LPS-induced human RA synovial fibroblastic E11 cells (67). Notably, the effects of pan-HDAC inhibitors in reducing inflammatory gene expression in RA-FLS were recapitulated by HDAC3 inhibition (68). It has been demonstrated that STAT1 and phosphorylated STAT1 levels are elevated in RA-FLS, contributing to inflammation (69, 70). Also, STAT1 hyperacetylation is a prerequisite for STAT1 dephosphorylation and inactivation (71). Notably, the genetic inhibition of HDAC3 attenuated IL-1β-induced STAT1 phosphorylation in RA-FLS, implicating a critical role of HDAC3 in RA-FLS activation and associated inflammation (68). However, the inhibition of HDAC3/6 did not affect the acetylation status of STAT1 in the presence of IL-1β in RA-FLS (68), suggesting a novel mechanism by which HDAC3 regulates STAT1 phosphorylation, warranting investigation.

Extracellular cold-inducible RNA-binding protein (CIRP) is a novel pro-inflammatory molecule involved in various inflammatory diseases. In patients with RA, increased CIRP levels are found in the serum and synovial fluid, and elevated CIRP levels in the synovial fluid correlate with disease activity (72). A recent study has reported that human CIRP induced the proliferation, migration, and invasion of RA-FLS and released IL-1β and IL-33 from RA-FLS (73). Moreover, the inhibition of CIRP significantly reduced the abnormal activation of RA-FLS and arthritis severity in adjuvant arthritis in rats (73). Per the same study, the knockdown of TLR4 inhibited extracellular CIRP-induced RA-FLS activation and HDAC3 expression in RA-FLS, suggesting a role of CIRP-TLR4-HDAC3 signaling in RA-associated synovial inflammation. Also, both genetic and pharmacological inhibition of HDAC3 suppressed extracellular CIRP-induced abnormal activation of RA-FLS *in vitro* and RGFP966 treatment attenuated arthritis severity of adjuvant arthritis in rats (73), implicating a crucial role of HDAC3 in RA-associated synovial inflammation. Therefore, further studies are highly warranted to establish the precise role of HDAC3 in RA pathology and the mechanism by which HDAC3 regulates synovial inflammation in RA.

Apart from RA-FLS, the other cell type that plays a crucial role in the RA pathophysiology is peripheral blood mononuclear cells (PBMC), which could release abnormal levels of inflammatory cytokines (74). A recent study postulated that before the development of synovitis in RA patients, systemic autoimmunity is initiated, resulting in cells such as monocytes from the peripheral blood infiltrating into the synovial tissue or joint fluid, causing inflammation (75). Also, the cytokines released from PBMCs can induce the differentiation of helper T cells towards Th1, Th2, Th17, and Treg cells, thereby modulating inflammation in RA (76). Consistent with the emerging role of epigenetic mechanisms in regulating cytokine release in RA (77), changes in HDAC activity in PBMCs from RA have been reported. However, there are inconsistencies between studies. For instance, a global increase in HDAC activity in PBMCs in RA and the efficacy of selective inhibition of HDAC3 in attenuating IL-6 release from RA PBMCs have been reported (78). On the contrary, another study reported a reduction in total HDAC activity and HDAC3 activity with an increase in total histone H3 acetylation in PBMCs from RA patients compared to healthy subjects (79). Despite these conflicting observations, the balance between HDAC and HAT activity was significantly altered in RA PBMCs, implicating further a potential role of histone acetylation in the pathophysiology of RA (79). Therefore, additional investigation is highly required to establish the precise functional role of HDAC3 and epigenetic mechanisms in PBMC-associated pathology in RA.

Osteoarthritis (OA) is another common form of arthritis in which HDAC3 plays a role. The expression of HDAC3 was shown to be higher in degraded cartilage compared to non-degraded cartilage. Furthermore, HDAC3 expression increased when primary human chondrocytes (PHCs) were stimulated with IL-1β (80), and the genetic inhibition of HDAC3 in PHCs augmented cartilage-specific genes and reduced the expression of a hypertrophy-related gene (80).

Overall, HDAC3 could be an efficient target to improve outcomes after RA and OA. However, further studies are highly warranted to elucidate the efficacy of selective HDAC3 inhibition and the mechanism by which HDAC3 regulates the development and progression of RA and OA.

## HDAC3 in cardiovascular inflammation

Atherosclerosis is the major underlying pathology of cardiovascular diseases (CVD), the leading cause of morbidity and mortality globally (81). Atherosclerosis is a chronic inflammatory disease arising from an imbalance in lipid metabolism and a maladaptive immune response. Macrophages play a key role in the progression and regression of atherosclerotic cardiovascular disease (82). Increased expression of pro-inflammatory cytokines, such as IL-1β, could regulate the expression of cholesterol efflux protein ABCA1 in macrophages, thereby promoting foam cell formation and development of atherosclerosis (83). Several studies have explored the therapeutic potential of HDAC inhibitors in CVD. Interestingly, myeloid-specific conditional deletion of HDAC3 shifted macrophages to an anti-inflammatory phenotype with improved lipid accumulation and plaque stability in a mouse model of atherosclerosis (84). Apart from regulating macrophage phenotype, HDAC3 plays an important role in regulating the adhesion of monocytes to the sites of inflammation (85). To this end, knockdown to HDAC3 attenuated TNF-α–mediated VCAM-1 expression in human primary endothelial cells (Human Umbilical Vein Endothelial Cells; HUVECs) and monocyte adhesion to the activated HUVECs. Also, in humans, HDAC3 was the sole HDAC upregulated in ruptured lesions and its expression inversely correlated with plaque-stabilizing TGF-β (84).

HDAC3 regulates endothelial function in normal physiology and pathology. Lentiviral-mediated knockdown of HDAC3 in endothelial cells reduced cell survival, suggesting that HDAC3 plays a critical role in endothelial cell survival *in vitro* (86). In a mouse model of type 2 diabetes mellitus (T2DM), HDAC3 activity, but not protein expression, was found to be increased in endothelial cells (87). Moreover, treatment with RGFP966 alleviated T2DM-associated endothelial dysfunction and the knockdown of Nrf2 abolished HDAC3 inhibition-induced endothelial protection in T2DM both *in vitro* and *in vivo* (87). Also, HDAC3 has been shown to regulate the expression of immune modulator galectin-9 in HUVECs (88). Notably, HDAC3 knockdown in endothelial cells reduced IFN-γ-induced expression of galectin-9, whereas overexpression of HDAC3 induced the interaction between IFN response factor 3 (IRF3) and phosphoinositol 3-kinase (PI3K) leading to IRF3 phosphorylation and galectin-9 expression (88). Evidence suggested that HDAC3 could serve as a scaffold protein facilitating PI3K/IRF3 interaction and regulating galectin-9 expression in endothelial cells (88).

Endothelial to mesenchymal transition (EndMT) contributes to multiple vasculopathies, including atherosclerosis, and facilitates the transition from vascular inflammation to plaque formation (89, 90). Of note, HDAC3 expression was upregulated in atherosclerotic plaque in a mouse model of atherosclerosis and regulated the induction of EndMT (91). Functionally, the pharmacological inhibition of HDAC3 in a mouse model of atherosclerosis reduced atherosclerotic lesions and inhibited EndMT, whereas the genetic overexpression of HDAC3 induced EndMT in HUVECs (91). Also, HDAC3 modulated the gene expression of IL-6, ICAM-1, and MCP-1 in HUVECs and the number of monocytes attached to HUVECs in the presence of inflammatory stimuli (91). These findings suggest a critical role of HDAC3 in vascular inflammation and the induction of EndMT.

Myocardial infarction (MI) is a common and life-threatening condition in which a blockage in the coronary artery leads to oxygen deprivation, injury, and cell death. The cell death can cause a high degree of inflammation as macrophages are recruited to the injury. In a rat model of ischemia-reperfusion injury, treatment with RGFP966 alleviated inflammatory response, oxidative stress, and injury in myocardial tissue, possibly by reducing the levels of cyclin-dependent kinase-2 (92), further implicating the efficacy of HDAC3 inhibition in reducing inflammation.

Though the aforementioned studies point out the potential of HDAC3 inhibition in alleviating cardiovascular inflammation, a contradictory finding documents an inverse association between the expression of HDAC3 and NF-κB/p65 in ox-LDL-induced HUVECs (93). As per the study, the overexpression of HDAC3 attenuated the levels of TNF-α and IL-1β in the arterial tissue in a mouse model of atherosclerosis (93). Due to this discrepancy between observations, additional investigation is necessary to validate the efficacy of HDAC3 inhibition for cardiovascular diseases.

## HDAC3 in pulmonary inflammation

While many studies reported a reduction in the proinflammatory response when HDAC3 was inhibited, some studies have indicated the inhibition of HDAC3 can augment inflammation. For instance, in chronic obstructive pulmonary disease (COPD), a pathological condition resulting from inhalation of air pollutants and cigarette smoke, pulmonary macrophages secrete a large and varied number of inflammatory factors. Using a model of human alveolar macrophages, it has been shown that acute cigarette smoke exposure is associated with reduced total nuclear HDAC activity and nuclear HDAC3 protein expression (94). Also, siRNA-mediated knockdown of HDAC3 in the *in vitro* model of alveolar macrophages augmented LPS-induced release of IL-1β and IL-8, possibly implicating a negative regulatory role of HDAC3 in inflammation, but mechanistic studies are yet to be conducted. Given the role of HDAC3 in modulating the acetylation status and the nuclear export of NF-κB, the study postulated that NF-κB signaling could be a possible mechanism by which HDAC3 regulates pulmonary inflammation (94). Also, Ergosterol treatment switched macrophage polarization to M2 phenotype with an increase in HDAC3 expression and a reduction in acetyl NF-κB/p65 in COPD models (95), suggesting that ergosterol-mediated protection of COPD is associated with HDAC3-mediated deacetylation.

Acute lung injury is characterized by damage to alveolar epithelial cells and capillary endothelial cells, causing refractory hypoxemia and acute respiratory distress syndrome. Nimbolide, a chemical constituent of *Azadirachta indica*, improved endotoxin-induced acute respiratory distress syndrome by inhibiting TNF-α mediated nuclear translocation of both NF-κB and HDAC3 (96). Furthermore, RGFP966 reduced the levels of proinflammatory cytokines in a model of inflammatory lung disease. Using an *in vitro* approach, the study also reported that the anti-inflammatory effects of RGFP966 are attributed to the modulation of NF-κB transcriptional activity but not NF-κB p65 acetylation or localization (97). Furthermore, Th2 cytokine-driven pulmonary inflammation was limited in mice lacking HDAC3 in macrophages (39), which implies that inhibition of HDAC3 can be targeted to attenuate lung inflammation.

Neutrophilic airway inflammation is associated with reduced total HDAC activity in blood monocytes (98), implicating a possible role of epigenetic mechanisms in the disease pathology. However, there was no change in HDAC3 gene expression levels.

Taken together, the current data implicate opposing roles of HDAC3 in pulmonary inflammation, with inhibition being either deleterious or beneficial. Further research using alternate approaches is necessary to validate the findings and elucidate the mechanism by which HDAC3 regulates pulmonary pathologies.

## HDAC3 in diabetic inflammation

HDAC3 has been implicated in playing a role in diabetic-related inflammation, which can interfere with insulin signaling and glucose homeostasis. *In-vitro* studies have shown that genetic knock-down of HDAC3 restored glucose-stimulated insulin secretion and reduced caspase-3 activity in beta cells in the presence of cytokines (99). Also, HDAC3 inhibition improved pancreatic β cell function and plasma glucose levels in a rat model of type 2 diabetes (100). Interestingly, increased HDAC3 activity and mRNA expression were observed in the PBMCs of type 2 diabetic patients in comparison with control subjects and HDAC3 activity positively correlated with proinflammatory markers, fasting plasma glucose, and insulin resistance (101). These findings suggest a critical role of HDAC3 in inflammation and other complications associated with diabetes.

Inflammation and apoptosis are the key mechanisms responsible for diabetic osteoporosis (102). In a streptozotocin (STZ) model of diabetes, HDAC 1 and 3 expression in femoral heads was found to be upregulated (103). Puerarin (PU), an isoflavone, improved STZ-induced blood glucose levels and osteoporosis with a reduction in inflammation and apoptosis in rats (103). Also, PU reduced STZ-mediated upregulation of HDAC1 and 3 expressions in femoral heads, suggesting a possible mechanism by which Puerarin (PU) improved diabetes-related complications. In line with this finding, inhibition of HDAC1/3 attenuated inflammation and cell death in fructose-treated cells (103). However, further studies need to be conducted to find which isoform of HDAC, among HDAC1 and 3, is responsible for PU-mediated effects.

RGFP966 has been shown to have beneficial effects in diabetic cardiomyopathy (DCM) in mice, causing a reduction in diabetes-induced cardiac oxidative stress, inflammation, fibrosis, hypertrophy, and insulin resistance (104). Notably, HDAC3 activity and phosphorylated extracellular regulated kinases 1 and 2 (ERK1/2), an indicator of cardiac hypertrophy, were upregulated in diabetic hearts (104). Additionally, the level of a nuclear ERK1/2 phosphatase, DUSP5 (dual specificity phosphatase 5), was decreased in diabetic hearts (104). Mechanistically, RGFP966 treatment augmented DUSP5 expression, modulated ERK1/2 signaling, and prevented DCM in mice (104).

Diabetes enhances the risk of stroke and its recurrence. RGFP966 treatment conferred protection against cerebral ischemia/reperfusion injury in diabetic mice by modulating brain oxidative stress, apoptosis, and autophagy (105). Also, RGFP966 treatment has been shown to have beneficial effects in some liver pathologies, particularly diabetes-induced liver damage. In diabetic mice, RGFP966 treatment reduced hepatic inflammation, fibrosis, and oxidative stress (106). These protective effects were associated with enhanced signaling of Nrf2 (106), an antioxidant and anti-inflammatory transcription factor. However, further studies are warranted to ensure the safe use of HDAC3 inhibitors for hepatic pathology, as liver-specific deletion of HDAC3 resulted in fatty liver in mice (107). Also, HDAC3 genetic deletion rescued palmitate-induced reduction in the expression of genes related to fatty acid oxidation in C2C12 myotubes, further implicating the role of HDAC3 in fatty acid metabolism (108).

## HDAC3 in allergic inflammation

Histone acetylation and deacetylation play roles in allergic inflammation (109). Of note, HDAC3 expression was upregulated in a mouse model of triphasic cutaneous anaphylaxis (triphasic cutaneous reaction; TpCR) (110). Moreover, HDAC3 regulated the expression of monocyte chemoattractant protein 1 (MCP1; a mediator of monocyte recruitment) and allergic skin inflammation *in vivo* (110). Besides, the suppressor of cytokine signaling 1 (SOCS1), a protein with contradictory roles in inflammation, regulated the expression of HDAC3 and allergic inflammation (111). Notably, antigen stimulation enhanced the expression of SOCS1, HDAC3, and HDAC6, in RBL2H3 basophilic leukemia cells (111). Furthermore, SOCS1 increased the interaction between high-affinity IgE receptor (FcϵRIβ) and HDAC3 in an antigen-independent manner, implicating a critical role of SOCS1/HDAC3 signaling in allergic inflammation (111).

The levels of hyaluronic acid (HA), a major component of the extracellular matrix, are elevated in allergic reactions *in vivo* and the increase in HA correlates with the influx of inflammatory cells (112). Despite the altered levels of HDAC3 in allergic response, it was postulated that HDAC3 may regulate allergic inflammation by modulating the production of low or high-molecular-weight HA (43). Furthermore, a study examining the role of HDAC3 in allergic rhinitis (AR) demonstrated decreased levels of multiple pro-inflammatory cytokines, and reduced allergic responses in mice upon RGFP966 treatment (113).

Altogether, various studies reveal a crucial role of HDAC3 in allergic pathologies. However, since the differences in the functional roles of HA of varying sizes are controversial, further studies are highly required to elucidate the pro or anti-allergic effects of high and low-molecular-weight HA and the mechanism by which HDAC3 regulates HA production and allergic inflammation.

## HDAC3 in renal inflammation

Inflammation plays a major role in chronic kidney disease (CKD), as it can lead to fibrosis and renal damage. It has been demonstrated that in a mouse model of kidney fibrosis, HDAC3 protein expression in the kidney was elevated and the deletion of HDAC3 via a genetic approach (CAG-Cre+) reduced the renal expression of TNF-α and fibrosis in a mouse model of kidney fibrosis (114). Also, in a rat model of hyperuricemia-induced fibrosis, the depletion of HDAC3 via a genetic approach blunted renal fibrosis (115). Altogether, the data implicate a key role of HDAC3 in renal fibrosis and associated inflammation. In line with this, treatment with RGFP966 attenuated kidney fibrosis in mice (114). Though the mechanism by which HDAC3 regulates fibrosis is yet to be defined, based on *in vitro* studies, it was postulated that hyperacetylation at Lys122 could reduce the transcription activity of NF-κB upon HDAC3 deletion (114), which further implicates a critical role of NF-κB acetylation in inflammatory disease conditions.

Acute kidney injury (AKI) and CKD are two distinct pathologies, but AKI can progress to CKD, characterized by various pathological events, including inflammation (116). However, the precise mechanism by which AKI progresses to CKD is largely unknown. In a mouse model of AKI-CKD, HDAC3 was found to be elevated in the kidney, and HDAC3 conditional deletion attenuated renal ferroptosis, and fibrosis (117). Consistent with this finding, RGFP966 treatment reduced renal ferroptosis and fibrosis in AKI-CKD mice, with a modulation in the expression of GPX4, a master regulator of ferroptosis, implicating a key role of HDAC3 in AKI-CKD transition (117).

## Conclusions and future directions

**Figure 1:**
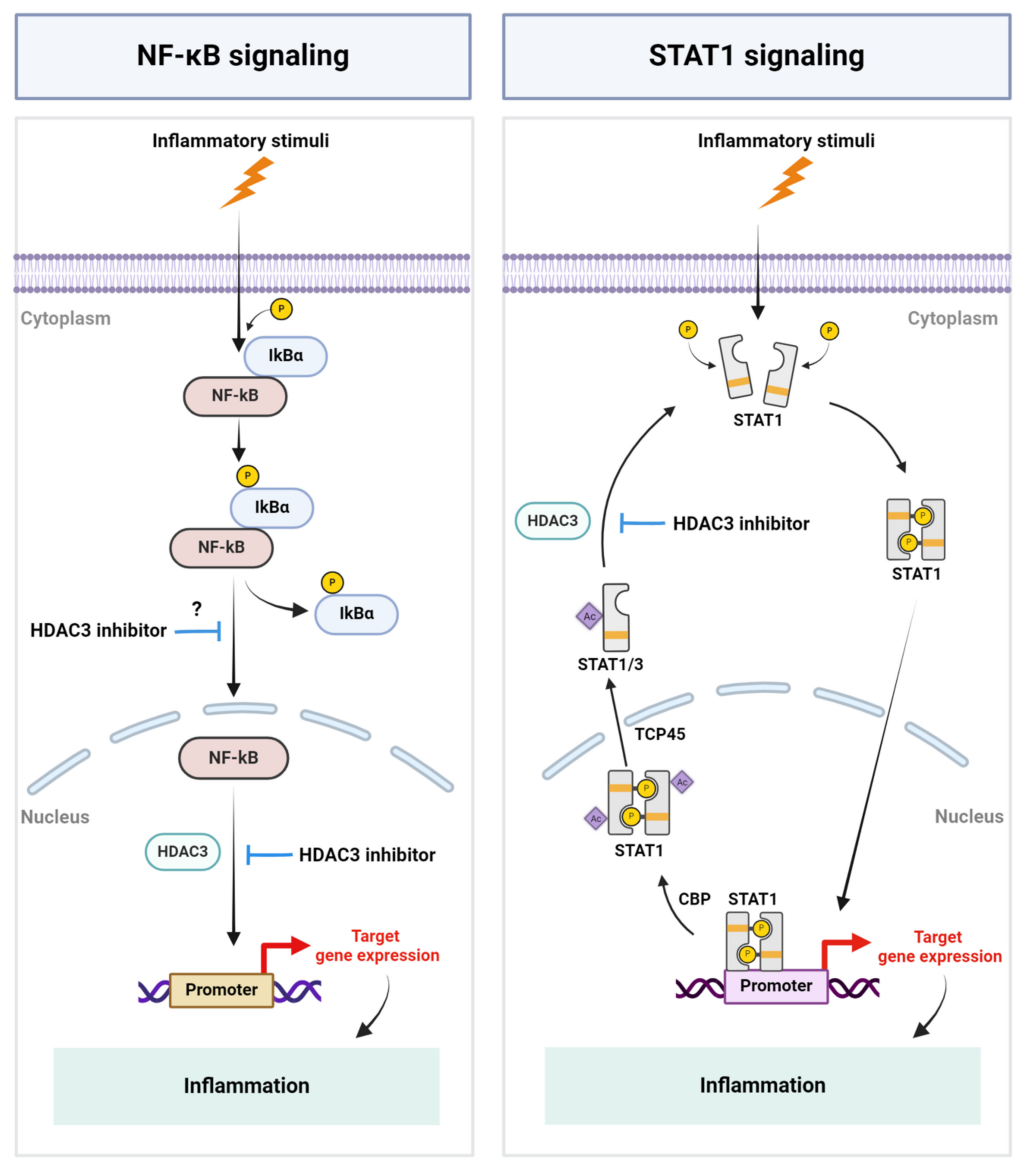
Schematic representation of key mechanisms by which HDAC3 regulates inflammation. Inflammatory stimuli cause the activation of the NF-kB signaling pathway and HDAC3-mediated NF-kB DNA-binding, resulting in the expression of NF-kB-regulated genes, including inflammatory genes. HDAC3 regulates STAT signaling by modulating the acetylation status of STAT1 and STAT3. P, Phosphate group; IkBα, I kappa B alpha; Ac, Acetyl group; CBP, CREB binding protein; TCP45, T cell protein tyrosine phosphatase 45. Created with BioRender.com.

HDAC3 has emerged as a pivotal regulator of a wide range of inflammatory conditions and has been demonstrated to play a role in immune cell differentiation and inflammatory gene expression. However, despite the emerging interest in targeting HDAC3 to modulate inflammation and disease pathologies, the precise mechanism by which HDAC3 regulates inflammatory gene expression profiles remains enigmatic. Mechanistic studies mostly employed in vitro approaches and focused on, and implied, to a large extent, the role of transcription factors such as NF-kB and STAT1 and their post-translational modification by HDAC3 in modulating inflammation (**Figure 1**). To gain further mechanistic insights, cell-specific functional studies using transgenic or conditional knock-out animals and *in vivo* studies employing unbiased proteomic and transcriptomic approaches are critical and required. Analyzing the enzymatic and nonenzymatic functions of HDAC3 and developing additional selective inhibitors or activators of HDAC3 could also be helpful in further defining its role in various pathological conditions. Despite some conflicting reports, the selective inhibition of HDAC3 has been demonstrated to play a beneficial role in various inflammatory pathologies. The selective inhibition of HDACs could also circumvent the off-target effects of pan-HDAC inhibitors. Notably, RGFP966 (10 mg/kg) selectively inhibited HDAC3 over other HDACs in mice (118), and its systemic administration (10 mg/kg) daily for 14 days did not induce significant toxic effects on the mouse brain and major organs (119), implicating its suitability for therapeutic purposes. However, clinical studies have yet to be conducted evaluating its safe use and efficacy in humans. Besides its therapeutic potential, HDAC3 activity or expression is altered in several pathological conditions, implicating its potential to serve as a diagnostic and prognostic marker of inflammation, warranting investigation. Overall, continued research into the interplay between HDAC3 and inflammation holds promise for advancing our understanding of inflammatory diseases and developing more effective treatment strategies.

## Author contributions

NW: Writing – original draft, Writing – review & editing. SK: Writing – original draft, Writing – review & editing. WL: Writing – original draft, Writing – review & editing. SS-R: Conceptualization, Funding acquisition, Project administration, Writing – original draft, Writing – review & editing.

## Glossary

**Table:**

ABCA1	ATP (Adenosine triphosphate)-Binding Cassette Transporter 1
AIM2	Absent in Melanoma 2
AKI	Acute Kidney Injury
AR	Allergic Rhinitis
cGAS	cyclic GMP-AMP synthase
CIRP	Cold-Inducible RNA-binding Protein
CKD	Chronic Kidney Disease
CNS	Central Nervous System
CVD	Cardiovascular Diseases
DAD	Deacetylase Activating Domain
DCM	Diabetic Cardiomyopathy
DNA	Deoxyribonucleic Acid
DUSP5	Dual Specificity Phosphatase 5
EndMT	Endothelial to mesenchymal transition
ERK1/2	Extracellular signal-regulated Kinase ½
FLS	Fibroblast-like synoviocytes
GSDMD	Gasdermin D
HA	Hyaluronic acid
HAT	Histone Acetyltransferases
HDAC	Histone deacetylases
HDAC3	Histone deacetylase 3
HDAC3/6	Histone deacetylase 3/6
HDACi	HDAC inhibitors
HUVECs	Human Umbilical Vein Endothelial Cells
IBD	Inflammatory Bowel Disease
ICAM-1	Intercellular Adhesion Molecule 1
IFN	Interferon
IL1β	Interleukin 1β
IL-6	Interleukin 6
IL-8	Interleukin 8
IL-33	Interleukin 33
iNOS	Inducible Nitric Oxide Synthase
IRF3	Interferon regulatory factor 3
LPS	Lipopolysaccharide
Lys122	Lysine 122
MCP-1	Monocyte Chemoattractant Protein-1
MI	Myocardial Infarction
NCoR1	Nuclear receptor co-repressor 1
NCoR2	Nuclear receptor co-repressor 2
NF-κB	Nuclear Factor kappa B
NLRP3	Nucleotide-binding oligomerization domain-like receptor 3
Nrf2	Nuclear factor erythroid 2-related factor 2
OA	Osteoarthritis
ox-LDL	Oxidized Low-Density Lipoprotein
P2X7R	Purinergic Receptor P2X7
PBMC	Peripheral Blood Mononuclear Cells
PHCs	Primary Human Chondrocytes
PI3K	Phosphoinositide 3-kinase
PPARγ	Peroxisome proliferator-activated receptor γ
PU	Puerarin
RA-FLS	Rheumatoid arthritis Fibroblast-like synoviocytes
RA	Rheumatoid arthritis
siRNA	Small interfering RNA
SIRT1	Sirtuin 1
SMRT	Silencing Mediator of Retinoic and Thyroid receptors
SOCS1	Suppressor of Cytokine Signaling 1
STAT1	Signal transducer and activator of transcription 1
STAT3	Signal transducer and activator of transcription 3
STAT5	Signal transducer and activator of transcription 5
STZ	Streptozotocin
T2DM	Type 2 Diabetes Mellitus
TGF-β	Transforming Growth Factor-β
Th1	T helper type 1
Th17	T helper type 17
Th2	T helper type 2
TLR	Toll-like Receptor
TLR4	Toll-like Receptor 4
TNF-α	Tumor Necrosis Factor-α
TpCR	Triphasic Cutaneous Reaction
Treg	Regulatory T cells
VCAM-1	Vascular Cell Adhesion Molecule 1.
